# Recent Strategies in Treatment of Pulmonary Arterial Hypertension, A Review

**DOI:** 10.5539/gjhs.v7n4p307

**Published:** 2015-01-25

**Authors:** Flora Fallah

**Affiliations:** 1 Shahid Sadoughi University of Medical Sciences & Health Services, Yazd, Iran

**Keywords:** Pulmonary Arterial Hypertension, recent strategies, treatment

## Abstract

Pulmonary arterial hypertension (PAH) is a disease characterized by an elevation in pulmonary artery pressure that can lead to right ventricular failure and death. The pulmonary circulation has to accommodate the entire cardiac output in each cardiac cycle and evolution has adapted to this by making it a low-pressure high-flow system. However, pathology can affect both the arterial and venous components of this system. Pulmonary venous hypertension mainly refers to diseases that result in elevated venous pressure and occurs mainly from mitral valve and left-sided heart disease. Standard treatment options include oral anticoagulation, diuretics, oxygen supplementation, and for a small percentage of patients, calcium channel blockers. Newer treatments include prostacyclin analogues, endothelin receptor antago¬nists, and phosphodiesterase type 5 inhibitors. This article reviews the current treatments strategies for PAH and provides guidelines for its management.

## 1. Introduction

In recent years, there has been outstanding progress in diagnostic information, pathophysiology and especially pulmonary hypertension (D’Alonzo et al., 1991). At the present time, we have a bulk of knowledge on molecular and cellular factors involved in causing disease. Breakthroughs of diagnostic methods and radiography have eased the diagnostic and separation of secondary factors. More importantly, the emergence of new and rather useful therapies has increased their fate and longevity. Many studies are directed at idiopathic formerly called primary pulmonary hypertension. Considering with significant progress in therapies, classification of diagnostic methods, it is essential to revision of the novel findings (D’Alonzo et al., 1991; Rubin, 1997).

## 2. History and Review Literature

The first report of pulmonary hypertension was mentioned by Ernest Roumberg in 1891 as arterial sclerosis was used by IbelIzera and was assumed to be caused by syphilis. It was not until 1940 when Esler Bernner reported the histopathology of 100 patients suffering pulmonary hypertension in which there was no syphilis evidence. Finally, Mr Drisdel et al mentioned a kind of pulmonary hypertension arterial vasculopathy associated with pulmonary hypertension, responding properly to Tolazoline. They used the notion of primary pulmonary hypertension. Thus, the cases with certain cause are called secondary pulmonary hypertension. Since 1973, based upon Consensus Conference, World Health Organization eliminated pathology basis for pulmonary hypertension. Ever since lung biopsy is not required for diagnosing primary pulmonary hypertension. The last classification of WHO was held with the presence of Consensus Conference on the basis of clinical findings of hemodynamic and due features (Rubin, 1997; ACCP, 2004).

Recent breakthroughs in diagnosis, survival and treatment of lung’s blood pressure make the change of its classification inevitable. Unlike the past, the cause assessment plays a pivotal role in the treatment. Accordingly, the last classification of WHO in 2003 is based on “cause”. In this classification, the patients suffering from pulmonary hypertension are divided into five groups. Patients in the first group are considered to be pulmonary arterial hypertension. And the rest are called pulmonary hypertension. When speaking of all five groups, the general notion of pulmonary hypertension (PH) is used (D’Alonzo et al., 1991). The last international summit was held in Dana point, the USA, in 2009. Accepting the previous classification, this summit modified some of the groups. Discussing the consensus classification of 2003, we will apply the modifications and talk about them.

## 3. Definition

Pulmonary hypertension (PH) is called a condition where the mean pressure of pulmonary artery is or greater than 25 mm Hg at rest or greater than 30 mm Hg in activity, provided that the pulmonary wedge pressure is under 15 mm Hg and pulmonary vascular resistance is equal or greater than 3 Wood/m^2^ (Adatia & Beghetti, 2009; Galie et al., 2009). In cases where the patient is at rest, pressure may be in the normal range and increases noticeably with activity. Therefore, an activity-oriented examination must be done on all suspicious patients.

The diagnosis of primary pulmonary hypertension is based on the known causes of its creation and the only acceptable people in this group are those with the exclusion of the known causes, only after necessary studies are done. Other cases of pulmonary hypertension have cause-oriented treatment (Adatia & Beghetti, 2009; Galie et al., 2009; Frost et al., 2011). It is better to put the primary and secondary notions aside as clinic, pathophysiology and treatments are different among secondary hypertension subgroups. For instance, patients suffering from both heart failure and obstructive pulmonary disease go to the secondary group whereas they have different pathophysiology and treatment (Frost et al., 2011).

## 4. Epidemiology of Pulmonary Hypertension

The outbreak of PH of secondary type depends on the prevalence of its causes in the community. For example, 20% to 30% of lung-disease patients can have degrees of pulmonary hypertension. In 30 to 40% of the patients, interstitial pulmonary hypertension has been reported (D’Alonzo et al., 1991).

**Figure 1:**
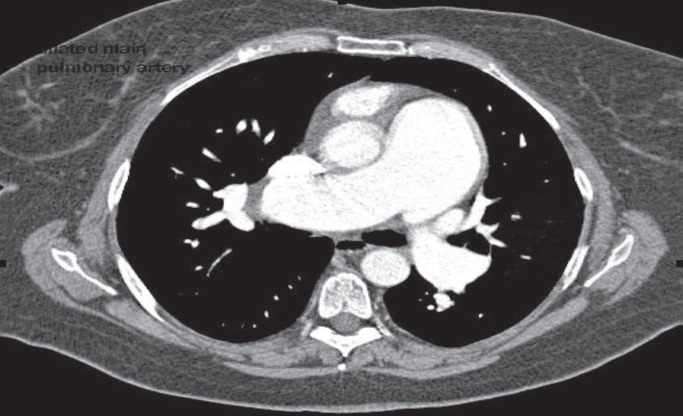
Chest CT scan showing a dilated pulmonary artery in idiopathic pulmonary artery hypertension

The prevalence of pulmonary hypertension among liver patients has led them to transplant liver in 5% to 20% and only a very small percentage of these patients have pulmonary similar to idiopathic forms. In a rather vast statistical study on the patients suffering chronic liver diseases, 0.6 to 0.73% of the patients were suffering idiopathic pulmonary hypertension. 9% to 19% of rheumatic patients have degrees of pulmonary hypertension. Among patients with SLE, scleroderma and more of crest syndrome and mixed connective tissue are 15%, 60% and 67% respectively. And it has rarely been observed among rheumatoid arthritis.

Idiopathic hypertension is a rare disease, occurrence rate of which is reportedly one to two per million. Accordingly, there are about 100 people annually for Iran’s 70 million population. Considering the longevity of patients’ 2-3 years (without treatment), there shall be about 200 to 300 patients suffering from pulmonary hypertension in Iran.

## 5. Pathophysiology and Pathology of Pulmonary Hypertension

According to Ohm’s law, factors affecting pulmonary pressure are pulmonary blood flow and the pulmonary vascular resistance. Accordingly, changes in right cardiac output (as the modifier of the “flow”), setting of pulmonary vascular resistance (the overall level of pulmonary vascular) and venous pressure of pulmonary system are the major factors of pulmonary hypertension (Humbert et al., 2006). This equation represents that pulmonary arterial hypertension depends on three factors:

**First**: right cardiac output

**Second**: pulmonary vascular resistance

**Third**: mean pulmonary artery pressure (usually mean pressure of right atrium).


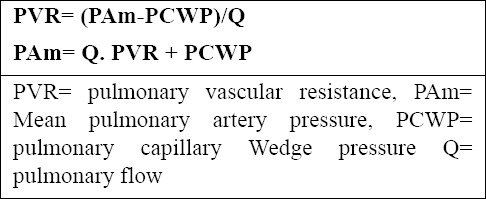


Accordingly, diseases such as atrial septal failure, ventricular septal failure and many congenital heart diseases such with the increase of blood flow (primary factor), diseases like pulmonary embolism, rheumatic diseases and pulmonary interstitial diseases through the overall decrease of vascular setting and increase of the pulmonary hypertension resistance (secondary factor) and diseases such as miteral stenosis, pulmonary venous occlusive and heart failure through the increase of venous pressure (third factor), all cause pulmonary hypertension (Humbert et al., 2006, Voelkel et al., 1997). It shall be noted that in many cases, there are a couple of factors involved in PH.

## 6. A Variety of Pulmonary Hypertensions on the Basis of the Last Venice International Consensus Conference in 2003

*First Group: Pulmonary Arterial Hypertension (PAH)*

Idiopathic Pulmonary Arterial Hypertension (IPAH)

IPAH is a rare disease with an incidence of roughly one or two individuals per 1 million yearly. Drawing the natural history of the disease is very difficult due to its rarity and similar diseases involved in its differential diagnosis. Several countries created an on-line system of the national register of the disease for overcoming this problem. This necessity is also being realized in Iran. Accordingly, since 1981 to 1985, 137 patients had been registered in France. This disease occurs in all age ranges, sexes and races. However, it’s a little more common among women (proportion is 1.7 to 1). Average age of the patients is 36.4 for both sexes and 9% of the patients are over 60 years old. Race prevalence was typical as that of population. These findings are also true for French, Japanese, Israeli and Mexican patients. In a French study on 674 patients suffering from PH, 39% was diagnosed as primary pulmonary hypertension. 80% of the patients during the functional diagnostic classes of 3 & 4 and the average test of 6 minutes had an activity of 325 meters (Giaid et al, 1993; Newman et al, 2008; Roberts et al, 1997).

**Table 1:** Risk factors and associated conditions for pah identified during the evian meeting (1998) and classified according to the strength of evidence

A. Drugs and Toxins
1. Definite
• Aminorex
• Fenfluramine
• Dexfenfluramine
• Toxic rapeseed oil
2. Very likely
• Amphetamines
• L-tryptophan
3. Possible
• Meta-amphetamines
• Cocaine
• Chemotherapeutic agents
4. Unlikely
• Antidepressants
• Oral contraceptives
• Estrogen therapy
• Cigarette smoking
B. Demographic and Medical Conditions
1. Definite
• Gender
2. Possible
• Pregnancy
• Systemic hypertension
3. Unlikely
• Obesity
C. Diseases
1. Definite
• HIV infection
2. Very likely
• Portal hypertension/liver disease
• Collagen vascular diseases
• Congenital systemic-pulmonary-cardiac shunts
3. Possible
• Thyroid disorders

## 7. Pulmonary Hypertension Treatment

### 7.1 Standard Treatments

Before introducing new treatments, the average life expectancy of the patients with pulmonary hypertension was 2.5 years (D’Alonzo et al., 1991; Robinovitch, 2008). The development of due studies and the introduction of new medicines have upheld the disease’s treatment. Medicines like oxygen, calcium channel blockers, digoxin and diuretics, long been used and called as standard or conventional medicines. This treatment has been adopted from previously existing treatments for chronic cardiovascular and respiratory diseases and upon the common pathophysiology in congestive failure of left heart, hypoxic of pulmonary obstructive disease, systemic blood pressure and considered as symptomatic treatment. None of these therapies are based on controlled studies. Some of them like diuretics for swelling, have been established on the reduction of swelling and its prescription and/or clinically prescribed on the basis of lab observation or autopsy in the increase of cardiac output (like digoxin). Medicines like anticoagulants are prescribed based on some uncontrolled retrospective and one introspective study on the patients with pulmonary hypertension. On the other hand, clinical response to diuretics is so much obvious in short-term that alleviate the need for a controlled study. Based upon the primary study on the patients with positive vasoactive, taking calcium channel blockers cause the fall of pulmonary pressure, pulmonary vascular resistance and the increase of the patients’ survival. However, the recent French study showed that only 6.8% of pulmonary hypertension patients are able to continue the treatment. Also, we should remember that only 50% of the patients responding to vasoactive test respond to the treatment of calcium channel blocker (13).

**Figure 2:**
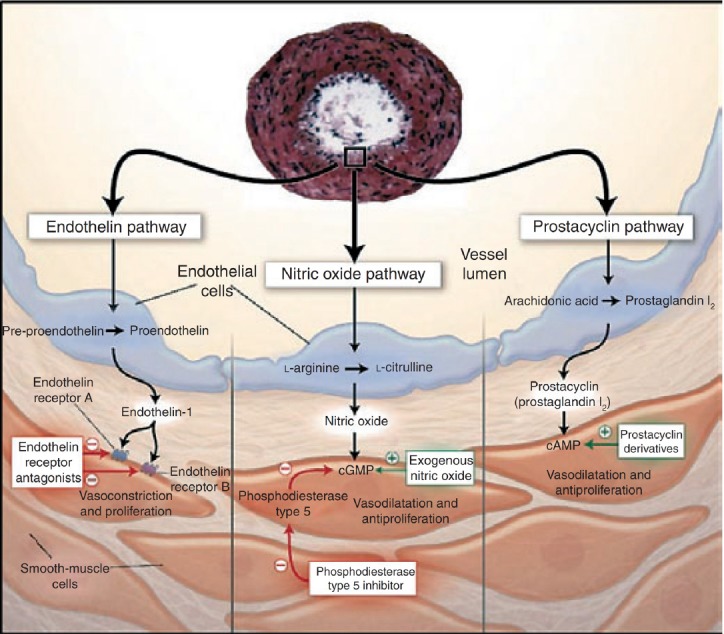
Targets for Current or Emerging Therapies in Pulmonary Arterial Hypertension

Targets for current or emerging therapies in pulmonary arterial hypertension. Three major pathways involved in abnormal proliferation and contraction of the smooth muscle cells of the pulmonary artery in patients with pulmonary arterial hypertension are shown. These pathways correspond to important therapeutic targets for the medications used to treat this condition: endothelin receptor antagonists, phosphodiesterase 5 inhibitors, and prostanoids. Plus signs denote an increase in the intracellular concentration; minus signs blockage of a receptor, inhibition of an enzyme, or a decrease in the intracellular concentration [Reproduced with permission from Humbert et al. (2004); All rights reserved].

According to the aforementioned information, prescription of the standard medicines is based on the identification of potential side-effects and symptomatic effects of the drug.

**A: Oxygen**

There is no controlled study to analyze the effect of oxygen. The basis of the treatment has been extracted from its impact on pulmonary obstructive patients. Anoxia affects pulmonary vascular heavily in terms of vasoreactive. In pulmonary obstructive patients, this effect has been carefully investigated (Humbert et al., 2001; Badesch et al., 2007; Ghofrani et al, 2004).

In the two controlled independent studies, the long-term effect of oxygen therapy has been investigated on pulmonary obstructive patients and evidence of right-heart failure. In both studies, the increase of patients’ life expectancy has been approved. When the pulmonary vascular resistance is high, the effect of oxygen therapy is less. However, it does not seem that oxygen therapy effect rises for the decrease of pulmonary vascular resistance.

According to above studies on pulmonary obstructive patients, oxygen therapy is prescribed with the same criteria as that of the patients suffering from pulmonary hypertension (Roberts et al., 2001). Many of these patients have hypoxia with activity on sleep or at rest. In these patients, hypoxia is caused by anoxia of secondary mixed venous and decreased cardiac output. Some patients suffering from pulmonary hypertension has sudden progressive hypoxia caused by sudden opening of the oval hole of the atrial wall and creating right to left shunt. Except for this group, patients’ hypoxia responds well to 2-6 liter oxygen per minute by nasal cannula (Badesch et al., 2007; Ghofrani et al, 2004; Roberts et al., 2001).

Often the existing guidelines suggest that all patients with pulmonary hypertension and plummet of arterial oxygen to less than or equal 55 mm Hg or plummet of oxygen saturation to less than or equal to 88% at rest, on sleep or in activity must take oxygen to the point that arterial oxygen pressure is always maintained above 90 mm (Ghofrani et al, 2004).

In patients who are diagnosed with chronic hypoxia, according to clinical or lab evidence such as hematocrit above 55%, clinical or strip evidence of right-heart failure are recommended to use oxygen with arterial pressure of less than or equal to 60. This group is usually rechecked in three months for hypoxia (Roberts et al., 2001). All patients with C gas diffusion of less than 60% shall go under oxygen therapy in activity or on sleep. It shall be noted that all these recommendations are based on the consensus of the experts of the field and without any controlled studies. The necessity of prescribing oxygen in patients with congenital heart disease and hypoxia still remains important. In some studies, giving oxygen to these patients particularly in children reduces the need for scientific bloodletting and clinical symptoms.

However, on sleep in patients with pulmonary hypertension, it is recommended to prescribe oxygen when arterial oxygen falls below 90% over 5% of sleeping time and regardless of the amount of loss. O starting oxygen on sleep, it is necessary to reexamine its quality. In most of these patients 2-3 liters per minute suffice by nasal cannula. The only exception is sleep apnea, which in the case of existence CP and BP are used. It is not recommended to routinely check the patients for sleep apnea. Even in the most severe form the disease, sleep therapy apnea will not cause significant improvement in pulmonary hypertension in any way. Sleep apnea, in any form or intensity, will not cause medium to severe blood pressure as the one observed with primary pulmonary hypertension. In patients with obesity hyper ventilation, oxygen is not sufficient alone and it is essential to use tracheostomy and ventilator (Badesch et al., 2007; Roberts et al., 2001).

Most of the patients and doctors are skeptical about taking oxygen and its usefulness. The truth is that besides considerable financial expenses and inappropriate systems of ambulatory oxygen therapy, it requires lifestyle changes which make its acceptation difficult. Basically, in our country, oxygen therapy attitude is negative among specialists whereas it is essential for lung obstructive and pulmonary pressure patients. It is the fear of its side-effects which basically saves no room for consumption amounts amongst the patients. In practice, when the subject is clear to patients, doctors have explained the benefits and they are realized in a few months, the patients continue the treatment.

**Figure 3:**
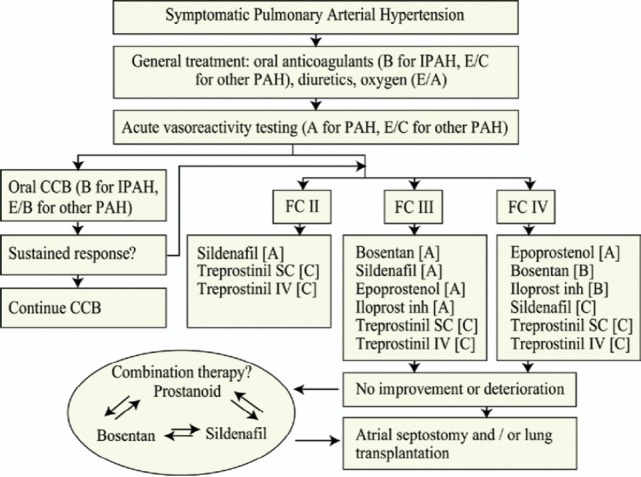
Treatment Algorithm for PAH Adapted From 2007 ACCP Guidelines

Letters following recommendations are based on a combination of level of evidence and perceived benefit: A=strong recommendation; B=moderate recommendation; C=weak recommendation. Recommendations with an E are based on expert opinion rather than clinical trial evidence. Ambrisentan was approved for functional class II and III pulmonary arterial hypertension (PAH) after these guidelines were published. ACCP=American College of Chest Physicians; CCB=calcium-channel blocker; FC=functional class; INH=inhaled; IPAH=idiopathic pulmonary arterial hypertension; IV=intravenous; SC=subcutaneous. Reproduced, with permission, from Badesch et al. (2007).

**B: Diuretics**

The long-term effects of diuretics have not been analyzed on the right-heart failures. However, its effect on the treatment of pulmonary hypertension patients with peripheral edema is generally acceptable. And it is applied to roughly all edema patients. Most of the patients show degrees of improvement and reduction of shortness of breath. This treatment is more effective I the case of salt and liquid restriction. Except for spironolactone, which has shown survival increase in a study on patients with severe left heart failure in class 3 and 4 in New York, the rest of the diuretics have been the focus of no studies. In above study, the consumption of spironolactone 12.5 mg in combination with inhibitors of angiotensin reduced two-year mortality from 46% to 35 % as to hospitalization rate. No one study has ever investigated this drug’s influence on right-heart failure and pulmonary hypertension. However, this medication is used with tube diuretics such as furosemide. It shall be noted ACE inhibitors useful in right-heart failure have no proved benefit on pulmonary hypertension. For kidney failure, spironolactone should not be used with creatinine above 2.5mg, potassium above 5 mg equivalent and the history of refractory hyperkalemia. Extreme care must be taken for patients with kidney failure, diabetic, elderly and those taking captopril or similar drugs simultaneously.

Amiloride in animal models has shown that it reduces pulmonary hypertension rising from hypoxia but there is no study on primary pulmonary hypertension. Its functional mechanism is associated with the prohibition of sodium- proton pump’s replacement and the prevention of alchemy intracellular which in itself causes the reduction of the proliferation of smooth muscles of vascular septum.

The right ventricular failure might require large amounts of diuretics. Some patients may need 600 mg of furosemide and 10 mg of bumetanide daily. Right ventricular function is associated with earlier times. Due to the continuity of the right and left ventricle (pericardium and common interventricular septum), excessive enlargement of the right ventricular can cause left ventricular diastolic failure and decreased cardiac output. Since the required hydrostatic pressure for creating pulmonary edema needs a wedge pressure of 40% plus a gradient ranging from mean pulmonary pressure and the wedges’ pressure, interstitial pulmonary edema is created with less wedge’s pressure. Also, a slight change in the patient’s liquids would result in severe cardiac changes. Thus, high doses of diuretics shall not be used.

Except in cases of cardiogenic pulmonary edema or acute exacerbation of hypoxemia, the optimal condition of fluid in patients suffering from pulmonary hypertension with right-heart failure must be maintained. This is a rather thin therapeutic network between maintenance of cardiac output and maintenance of blood oxygen with clinical symptoms of tissue perfusion.

Patients with severe right-heart failure and reduced cardiac output, passive congestion of the liver and swelling of the intestinal wall will appear in them. And as a result of a rise in liver tests and similar clinical conditions- due to the release of bacteria from the lining of the inflamed colon- dilated and ischemic are caused. Abdominal pain and tenderness and positive occult blood in excretion are observed without the negative hot bacteria, which might be fatal. In this case, proper treatment may include fluid therapy or diuretic medication. Depending on the circumstances we are facing, low dose of Dobutamine or dopamine, inhaled prostacyclin and antibiotic covering intestinal microbes must be considered.

Orthostatic hypertension means cutting down on intravascular size and diuretic. Concern of low pressure and prescription of insufficient dose of diuretic cause high swelling and worsening of the patient’s clinical situation. The maintenance of serum potassium is of high significant given the various consumptive drugs and shall be monitored. Palpitations, arrhythmias or syncope strokes may be caused by pulmonary hypertension and right ventricular failure. However, we should not ignore electrolyte imbalance and blood levels of digoxin.

Many pulmonary hypertension therapies such as calcium channel blockers, Bosentan, Sytalsntan and Prostacyclins create inflammation. Dose adjustment of diuretics usually solves the problem. Other inflammation therapies are: raising feet at certain times, wearing compression varicose socks and if necessary, substitution of the drug or coping with it.

**C: Digoxin**

Cardiac glycosides such as digoxin cause the increase of the contractile strength of the heart through blocking adenosine triphosphate sodium-potassium within the wall of the heart muscle cells. It has been shown on patients with right heart failure that a digoxin injection increases the cardiac output and decreases the circulation of norepinephrine. In a study on the patients with pulmonary obstructive disease and right heart failure, digoxin use had no effect on right heart contractility. Long-term consumption of digoxin has had no effect on mortality, morbidity and life expectancy of pulmonary hypertension patients with right ventricular failure. In a study on patients with 2 and 3 New York class and 2-5 year digoxin therapy only reduced the rates of hospitalization associated with heart failure with no effect on the patents’ morality. Currently digoxin is recommended to patients suffering from pulmonary hypertension and right ventricular failure with heart rhythmic disorders such as multifocal atrial tachycardia and atrial-flutter or atrial fibrillation. Some experts of the field recommend that the patients using calcium blockers should take digoxin to overcome its negative entropic effect (Barst, 2004).

Since most of the patients are diuretics simultaneously, it is essential to pay a special attention to blood level of digoxin and electrolytes. Paroxysmal tachycardia with changeable atrioventricular block is well-known arrhythmias. With the reduction of renal clearance, digoxin blood level increases. Digoxin usually starts with daily and orally dose of 0.25 mg to 0.125 mg and blood level is measured after a week. Among elderly, patients with renal disorder or light weight, a lower dose (0.125 mg every other day) is used. It is suggested that blood level be maintained at the range of 0.5- 1 ng. When there is no hypomanesium, hypokalemia hypothyroidism, below 2 nm toxicity cannot be observed. Patients suffering from coronary artery and chronic coronary syndrome, digoxin shall not be used due to arrhythmias risk and heart attack. Also, simultaneous use of clarithromycin, Amiodarone, Itraconazole and many more drugs can increase serum level of digoxin (Barst, 2004).

**D: Anticoagulants**

There is currently no prospective randomized controlled study representing evidence against using anticoagulants. Anticoagulants usage is accepted for pulmonary hypertension unless there is a contradiction to its use (Johnson et al., 2006; Badesch et al., 2004). Pulmonary hypertension patients may have an inactive lifestyle. Venous congestion, stasis (due to the high pressure of the right atrium) and low flow of blood in the pulmonary circulation and systemic (due to a fall in cardiac output) subject them to deep vein thrombosis. Pulmonary vascular bed is severely restricted in these patients and pulmonary embolism can be fatal due to hypoxia effects.

There is a lot of evidence that patients with primary and secondary pulmonary hypertension have a high tendency to form clots due to endothelial disorder, platelet activation and plasma proteins involved in coagulation and fibrosis. The evidence for increasing coagulation is obvious in the primary hypertension and the form arising from unresolved emboli. Some of these predisposing factors have shown that it can be modified using special treatments such as Bosentan and prostacyclin. Retrospective and some controlled studies suggest that warfarin consumption can increase life expectancy of pulmonary hypertension patients. This issue is specifically evident in hypertension rising from unabsorbed emboli. Because of the many clinical, hemodlynamic and histopathologic similarities of the patients with primary hypertension, this treatment is also acceptable for the other cases of pulmonary hypertension (Johnson et al., 2006; Badesch et al., 2004).

There is no human study on the type of the anticoagulant therapy. However, warfarin is often used for convenience. But animal studies show the extra effect of heparin. Heparin can prevent pulmonary high pressure and right ventricular hypertrophy in animal models. This effect may be from the inhibition of platelet growth factor and/or the impact on vascular smooth muscles of the lungs. So it may be possible someday in the future heparin compounds play a greater role in the treatment of pulmonary hypertension (Badesch et al., 2004; Broberg et al., 2007).

In practice, warfarin is intended to be used this way. And in most of the centers ENR value used is 1.5- 2.5. But, the target value is 2-3 in Europe. In the following conditions, however, it is better to be maintained above 2:

- Any disease which is in need of anticoagulation for reasons like atrial fibrillation, chronic embolism, thrombosis of the deep venous pelvic artificial valve.
- Patients with deep venous thrombosis history.
- Patients with the history of unabsorbed embolism but their perfusion scan is defected of sub-segment or show sub-segment activity decrease.
- Patients with cerebral ischemic stroke or transient strokes of cerebral ischemic without left to right shunt.

The role of aspirin and other antiplatelet drugs such as clopidogrel has not been studies properly on pulmonary hypertension.

In case of medium to high clinical suspicion to pulmonary hypertension and echo showing lung pressure over 60, anticoagulant can be started as the patient is being more examined. When an invasive bleeding action as surgery is required, warfarin can be stopped 5 to 7 days earlier without the need for heparin use unless it is required for another reason. When ENR is below 1.5, prophylaxis of clots must be prescribed for patients before surgery or hospitalization.

### 7.2 Specific Treatments

**A: Calcium Channel Blocker**

**Vasoactive Test**

Pulmonary hypertension feature is the rise of pulmonary vascular resistance. The increase of right ventricular coefficient (volume of the pumped blood by the opposite pressure) against the increased pulmonary resistance are considered to be the cause of dilatation, hypertrophy, and eventually right ventricular failure. It is an attractive idea to reduce resistance using pressure-lowering drugs, especially the basic and clinical studies on animals show the reduction of vasodilator mediators and the raise if increasing mediators among patients. Clinical studies suggest that reduction of pulmonary hypertension with pulmonary vasodilator drugs (calcium channel blockers, intravenous prostacyclin, inhaled anu gas, intravenous adenosine or inhaled Iloprost) in patients with PH will have a better prognosis when high dose of calcium channel blockers is used. This test is called vasoreactive and necessary to be done on all aforementioned patients except for patients with left ventricular failure and those who have received reduction output. If the test is positive, the patient is called responsive and should be put on long0term treatment of calcium channel blockers (Rich et al., 1992; Sitbon et al., 2005; Barst et al., 2004).

**Figure 4:**
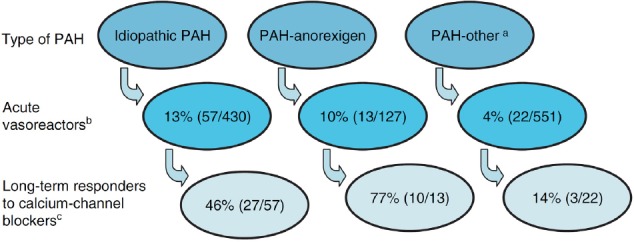
Breakdown of long-term responders to calcium channel blocker (CCB) monotherapy amongst those who are acutely vasoreactive, by type of PAH (data adopted from Sitbon et al. (2004) ^a^ Other includes PAH associated with connective tissue disease, veno-occlusive disease, pulmonary capillary haemangiomatosis, human immunodeficiency virus, portopulmonary hypertension, familial and congenital heart defects. ^b^ Acute vasoreactors were defined by a fall in both mean pulmonary artery pressure and pulmonary vascular resistance>20%. ^c^ Long-term responders were defined as those being in functional class I or II after at least 1 year on CCB monotherapy.

So, respondent is called someone showing over 10 mm Hg fall in pulmonary mean pressure. About 10 to 15 % of the patients have a positive test. This value is due to less embolism and scleroderma in hypertension patients and in this latter group, the role of the test is unclear. There are people who are not at first responsive and become in next sessions. The recent information suggests that at the end only 6.7% of the patients take advantage of this therapy. For this reason and other therapies, the number of the proponents of the test has decreased. However, it is still part of the diagnostic standard method for primary pulmonary hypertension patients (Rich et al., 1992; Sitbon et al., 2005). The initial object of the test is to take decisions on whether to give or not to give calcium channel blockers and it shall not be used with patients suffering from left-heart failure and the exacerbation of the right ventricle failure as it may lead to deterioration of oxygen and pulmonary edema. With the advent of the conditions above, one shall avoid vasodilator drugs and the prescription of morphine, intravenous nitroglycerin and furosemide is necessary. However, low dose and cautious prescription of pulmonary vasodilators such as prostacyclin is alright. Some suggest that intravenous Nitroprusside is used in these circumstances so that pulmonary edema would decrease with systemic hypertension and left-heart cardiac output. In patients suffering from Neoalkoziyo, vasoreactive test can lead to severe and lethal pulmonary edema. In these patients, even low dose of prostacyclin (2 ng kilogram per minute) can lead to death.

Nitric oxide gas or inhalation Iloprost, intravenous adenosine or prostacyclin can be used to test vasodilation without problems. However, drugs of calcium channel blocker shall not be used as they are long-acting ones and can lead to systemic severe hypertension and hypoxemia deterioration. This reaction is particularly evident in Nanrspandrha. For this reason, risk of pressure fall and even death, these drugs shall not be used in patients who have not been tested for vasoreactive test.

Patients responsive to the test can take calcium channel blockers’ therapy if there is no restriction on the part of the hospital. And the increase of the dose happens as outpatient people. Drug selection is done based on the heartbeat at rest; Dilitazem and Nifedipine are used for over and lower than 100 in minute respectively. Verapamil shall not be used because of the vasodepressive effects. Amlodipine is particularly used when there is a kind of intolerance to other drugs (increased swelling, hypertension, too high or low heartbeat). Target dose is different for calcium blocker drugs. Nifedipine and Diltiazem in three daily doses and amlodipine in two daily doses are prescribed. Outpatient therapy starts with 20 mg of Nifedipine and 60 mg of Diltiazem and it is increased gradually to the maximum of 240 mg and 720 mg for other above drugs during 6-12 weeks. Amlodipine maximum dose is 5 mg twice a day (Sitbon et al., 2004; Sitbon et al., 2005) (Table 2).

**Table 2:** Drug dosages in some calcium channel blocker

Description	Standard Max	Starting Dose and Daily Division	Type of Drug
Only 6.8% of the responsive patients take advantage of the therapy at the end and half the patients need another specific drug during a year	240 mg	20mg, three times	Nifedipine
	720 mg	60mg, three times	Diltiazem
	10 mg	Quarter of a tablet, three times	Amlodipine

**B: Prostanoids**

Different types of Prostanoids formulations are used in the treatment of pulmonary hypertension. They include Epoprostend, intravenous prostacyclin, subcutaneous and intravenous Treprostinil and an inhaled form called Iloprost. Oral form of the drug is called Beroprost and is still in the review process (Ghofrani et al., 2003; Hoeper et al., 2003).

Epoprostenol: most of the studies have been carried out on this drug intravenously. Hemodynamics improvement, functional capacity increase, and survival increase are all observed in the pulmonary hypertension patients when these drugs are taken. However, for the rest of the patients of the first group, there are only capacity increase and improvement of hemodynamics parameters (Hoeper et al., 2003; Olschewski et al., 1999).

In a random study on 81 patients in two therapeutic groups of standard and Epoprostenol, the group which was treated with this drug showed improvements on life quality indices, the mean reduction of pulmonary blood pressure, reduction of pulmonary vascular resistance and action ability (measured by 6 min activity test). 8 patients died during the study period all belonged to standard therapy group.

Now, this drug is the first choice of the patients from class 4. In the USA, most insurances confirm this treatment for patients with primary pulmonary hypertension rising from connective tissue diseases, liver patients, AIDS, anorexia drugs and congenital heart patients provided that the following items are taken into consideration:

- Despite medical and surgical treatments, hypertension advances.
- Lung pressure would be 25mm Hg at rest and 30 mmHg in activity.
- Pulmonary hypertension symptoms are present.
- Vasoreactive test is not positive or the therapy of calcium channel blockers is a failure.

Even the patients who are in other classes of WHO- but it is proved that lung pressure is high and disproportionate to the underlying disease- Epoprostenol prescription is covered. This rule is true for other drugs among advanced patients. This shows that the above drug with alternative intended usages of outstanding effects can be fully covered by the insurance. High costs and consumptive method are two major pitfalls on the prescription of the drug. In the meantime, the absences of a custodian for pulmonary hypertension and its inaccessibility have doubled the problem.

In practice, Epoprotenol starts as continuous intravenous injection with the presence of artery infusion pumps with 1-2 mg kg/min and increases each week or two until the results or side effects turn up. In case of abnormal increase of cardiac output, the dose is reduced. Significant effects include jaw pain, joint pain and diarrhea. Other important problems have to do with the infusion pump which might realize as thrombosis, pump dysfunction, infusion stop or intravenous infection.

Terprotinil, under the name of Remodulin, is used as continues intravenous or subcutaneous injection. It is used in the primary hypertension group and has effects like that of Epoprostenol. There is no study comparing these two together. Of advantages of Terprostinil, we can refer to subcutaneous method (which is painful), longer half-life (which prevents any life-threatening event happening in Epoprostenol, in case of drug use stoppage) and the lack of the need for a fridge to maintain. When the patient intends for the above benefits, it can be used in the first place or used as an alternative to Epoprostenol (Ghofrani et al., 2003; Hoeper et al., 2003; Olschewski et al., 1996).

Iloprost is used through inhalation 6 to 10 times daily in 3 and 4 class of the disease.

**C: Antagonist Receptors of Endothelin**

Endothelin 1 is a kind of highly strong retractor of pulmonary vascular, mitogen and stimulator of the growth of endothelia cells, smooth muscles and fibroblasts within the vessel walls. In the lungs of primary and first group of pulmonary hypertension patients including scleroderma and shunt cardiac patients, a sharp increase of Endothelin 1 is observed. There are two Endothelin -1 known as A and B. antagonists of Endothelin 1 for Endothelin A, has entered the therapy of pulmonary hypertension patients selectively or non-selectively since 15 years ago. These drugs improve the patients’ dyspnea, hemodynamic (including cardiac index, pulmonary pressure and pulmonary vascular resistance) and physical ability (Rubin et al., 2002; Giaid et al., 1993).

The leading drug is Bosentan (trochlear), the utmost benefit of which is being oral. This drug, the non-specific antagonist of Endothelin receptors, improves the patients’ performance and hemodynamic. And time would put off their symptoms’ exacerbation. patients’ mortality rate would decrease with these drugs. The most important side effect is liver toxicity associated with high dose and happens more with higher doses. Thus, it is necessary to check the liver enzymes during the treatment (Sitbon et al., 2005; Benza et al., 2006). Also, this is heavily teratogenic, so effective contraceptives must be employed when taken by women. It is forbidden for moderate to severe hepatitis patients and along with cyclosporine and glyburide drugs. Specific drugs for Endothelin A1 receptors including Sitaxentan and Ambrisentan have been introduced and studies. Ambristentan bears the approval of the USA Food and Drug Administration among others. It has been proved that these drugs increase performance, hemodynamics, life expectancy and life quality of the patients. Hepatitis is also observed in this group of drugs so it is essential to check the liver function during the treatment. Ambrisentan has got less drug contact. In Iran, the most restrictive factor for using these drugs is their incredibly high costs and despite availability, only a few can properly use it. Bosentan usually starts with 62.5 twice daily and continues with 125 mg daily after few weeks. One can make sure of the efficiency and continuation of the drug after 16 weeks. It is essential to do liver tests before and after the therapy (Giaid et al., 1993; Sitbon et al., 2005)

**D: Phosphodiesterase Inhibitors**

PDE inhibitors 1.3 and 5 are probably effective in the treatment of pulmonary hypertension. Tadanafil, Vardenafil and sildenafil are of this group. Among them, sildenafil, a cyclic GMP blocker, is type 5 due to phosphodiesterase taken orally (Galie et al., 2005). One can prolong the effects of nitric oxide vasodilator. This drug causes the hemodynamic improvement of the first group of pulmonary hypertension patients and improves their activities. And within the laboratory samples, this drug not only does not prevent the fall of pulmonary blood pressure but also stops its progress. But, its influence is not approved on other various pulmonary hypertensions. It may, however, affect the hypertension a rising from pulmonary obstructive diseases, embolism and going to mountains. This drug has the license of use for pulmonary hypertension patients of class 2 to 4 in Europe and America and it can be used as a combination therapy with Prostanoids or Bosentan. It seems like the influence of various types of PDE-5 blockers such as sildenafil, Tadanafil and Vardenafil is different on this disease and it is only sildenafil affecting pulmonary vascular bed. These drugs are different at the beginning of the effect, in being selective to pulmonary vascular and affecting oxygenation. This shall not be used with nitrate medications and shall be used cautiously due to its highly interaction with other drugs. In hypertensions caused by the increase of pulmonary venous pressure such as PVOD (like other special drugs of PH), this drug shall not be used. It can be used in pregnancy category B and is not clear in breastfeeding. It is essential to reduce the dose for renal and liver failure. So the measurement of basic creatinine is necessary by the starting time. It usually starts with 25 mg three times daily and can be increased vigilantly by 300 mg per day (Galie et al., 2006).

### 7.3 Other Treatments

There are other therapeutic groups which are being examined. Serotonin receptor antagonists and serotonin transporter blockers, rho-kinase blockers, vasoactive intestinal peptide (VIP), statins, tyrosine kinase inhibitors (TKLS) are drugs which might be used in the treatments of variety of PH in the future. However, they are mostly being used in studies and wherein there is no reliable clinical study.

**A: Combination Therapy**

There are a few studies on the harmless and effective therapies of Bosentan, Epoprotenol or Tereprostnil. The efficiency of Bosentan with sildenafil has been observed among the patients suffering from primary pulmonary hypertension and secondary form of scleroderma and it seems to have greater effect than mono therapy. However, more studies are required to analyze the role of these therapies on the patients’ future. Studies on the combination of Bosentan and Iloprost bear contradictory results and seem to be ineffective, there are some small-scale studies representing better results of the combination of Iloprost and sildenafil (Johnson et al., 2006; Simonneau et al., 2005). In recent years, most of the studies have focused on combination therapy to obtain a greater therapy influence. The most common method of combination therapy is to add the second drug with a different effect to the prior dug when the patient has severe symptoms with the former one or doesn’t take advantage of it. The other method is that we firstly suppose that a given drug is ineffective so the therapy shall be started with two or more drugs. The selection of the type of the drugs depends on each center’s experiences (Johnson et al., 2006).

The newer treatments might change the existing drug classes (new antagonists of Endothelin or newer drugs of Prostanoids) or aim the other factors affecting pulmonary hypertension. Our knowledge increase about causes of pulmonary hypertension would open new avenues. These drugs may belong to antagonist group of vascular receptors such as serotonin, vasoactive intestinal peptide (VIP) or cell anti proliferation drugs like Imatinib, statins and gene therapy (P38 MAP kinase inhibitors) (Johnson et al., 2006; Hoeper et al., 2005).

**B: Atrial Septostomy**

The idea of connecting right and left atria as a therapy originates from the fact that patients suffering from idiopathic pulmonary hypertension, having an open oral hole and those who are Eisenmenger, live longer. Linking the atriums in these patients with surgical or non-surgical procedure removes or decreases the pressure on the right ventricle, increases the filling of left ventricular and cardiac output. Creating a hole in the atrial septum has been performed with patients having various syncope or suffering from severe right heart failure. Loss of oxygen from creating a right-left shunt is compensated by better systemic blood circulation and ultimately oxygen delivery might be increased by 21%. Unfortunately, however, the mortality rate of this procedure is 15-20%. And it is difficult to predict who benefit this therapy. This therapy might be applied to patients suffering from pulmonary hypertension, right ventricular failure and those who have the clinical evidence of the decrease of systemic blood circulation such as syncope originating from the defect of left heart filling. It seems that patients with advanced pulmonary hypertension, the increased right atrial pressure (above 20 mm Hg), highly low cardiac output and arterial oxygen saturation less than 80% are at greater risk of death. Elderly and renal failures are also effective factors of the disease.

**Table 3:** Combination Therapy in PAH

Combination	6MWD Improvement* (m)	Other Evaluations
Bosentan _ inhaled iloprost STEP trial (n _ 67)	Peak†: 26 m (p _ 0.05) Trough: no difference	Improved functional class, time to clinical worsening, post-inhalation hemodynamics (p _ 0.05)

COMBI trial (n _ 40): unblinded randomized	No difference	No difference functional class, time to clinical worsening

IV epoprostenol _ oral therapy BREATHE-2 trial (n _33)	No difference	Trend toward greater improvement in PVR (p _ 0.08)

PACES trial (n _ 267) epoprostenol _ sildenafil	26 m (p _ 0.05)	Improved hemodynamics, time to clinical worsening (p _ 0.05)

Oral therapy _ inhaled treprostinil TRIUMPH-1 trial (n _ 235)	Walk improvement reported as positive (press release); results expected 2008	

In adequately powered studies, combination therapy has led to improvement in functional class, hemodynamics, and exercise capacity.*Improvement is placebo corrected. †Peak refers to post-inhalation, while trough was pre-inhalation. BREATHE-2 = Bosentan Randomized Trial of Endothelin Antagonist Therapy; COMBI = Combination Therapy of Bosentan and Aerosolized Iloprost in Idiopathic Pulmonary Arterial Hypertension; IV = intravenous; PACES = The Efficacy and Safety of Sildenafil Citrate Used in Combination With Intravenous Epoprostenol in PAH; PVR = pulmonary vascular resistance; STEP = Iloprost Inhalation Solution Safety and Pilot Efficacy Trial in Combination with Bosentan for Evaluation in Pulmonary Arterial Hypertension; TRIUMPH-1= Clinical Investigation Into Inhaled Treprostinil Sodium in Patients With Severe PAH.

## 8. Conclusion

The diagnosis and management of PAH has undergone a dramatic transformation in the last decade. Newer therapies developed on the background of a greater understanding of the molecular pathogenesis of the condition have entered routine clinical practice and have improved patient survival and well-being. Moreover there are still a large number of experimental studies taking place and there is great hope that it will not be long before more treatments are made available for these patients. As new treatment strategies appear on the horizon, clinical trials should consider investigating the mechanisms of treatment success or treatment failure by comparing treatment responders and non-responders, as is currently the novel approach developed for cancer treatment trials. PAH should be viewed as a cardiopulmonary system disease, which is based on the paradox of apoptosis, apoptosis-resistant growth of phenotypically abnormal cells and angiogenesis in the lungs, and apoptosis and capillary loss in the heart. Thus, new therapeutic strategies should consider that drugs originally designed to tackle angiogenesis in PAH lungs could potentially have a profound, negative impact on the failing RV.
